# Desalted Duck Egg White Peptides Promote Calcium Uptake and Modulate Bone Formation in the Retinoic Acid-Induced Bone Loss Rat and Caco-2 Cell Model

**DOI:** 10.3390/nu9050490

**Published:** 2017-05-12

**Authors:** Tao Hou, Yanshuang Liu, Nikolai Kolba, Danjun Guo, Hui He

**Affiliations:** 1 College of Food Science and Technology, Huazhong Agricultural University, Wuhan 430070, China; mrhoutao@webmail.hzau.edu.cn (T.H.); ysliu@webmail.hzau.edu.cn (Y.L.); guodanj@webmail.hzau.edu.cn (D.G.); 2 Key Laboratory of Environment Correlative Dietology, Ministry of Education, Huazhong Agricultural University, Wuhan 430070, China; 3 USDA-ARS, Robert W. Holley Center for Agriculture and Health, Cornell University, Ithaca, NY 14850, USA; nk598@cornell.edu

**Keywords:** desalted duck egg white peptides (DPs), retinoic acid, alendronate, transient receptor potential vanilloid 6 (TRPV6) calcium channel

## Abstract

Desalted duck egg white peptides (DPs) have been proven to promote calcium uptake in Caco-2 cells and rats treated with a calcium-deficient diet. The retinoic acid-induced bone loss model was used to evaluate the effect of DPs on calcium absorption and bone formation. Three-month-old Wistar female rats were treated with 0.9% saline, DPs (800 mg/kg), or alendronate (5 mg/kg) for three weeks immediately after retinoic acid treatment (80 mg/kg) once daily for two weeks. The model group was significantly higher in serum bone alkaline phosphatase than the other three groups (*p* < 0.05), but lower in calcium absorption rate, serum osteocalcin, bone weight index, bone calcium content, bone mineral density, and bone max load. After treatment with DPs or alendronate, the absorption rate increased and some serum and bone indices recovered. The morphology results indicated bone tissue form were ameliorated and numbers of osteoclasts decreased after supplementation with DPs or alendronate. The in vitro study showed that the transient receptor potential vanilloid 6 (TRPV6) calcium channel was the main transport pathway of both DPs and Val-Ser-Glu-Glu peptitde (VSEE), which was identified from DPs. Our results indicated that DPs could be a promising alternative to current therapeutic agents for bone loss because of the promotion of calcium uptake and regulation of bone formation.

## 1. Introduction

Osteoporosis, a metabolic disease of the bones, is becoming a common world health problem according the WHO [1]. There is a negative imbalance between bone formation and bone resorption resulting in bone loss and the etiology can be multifactorial, with contributions from diet and lifestyle, genetics like sex and ethnicity, and other medical conditions [2]. Postmenopausal osteoporosis accounts for a large proportion of osteoporosis in patients. In women, this imbalance is induced by the increase in the activation frequency of new remodeling units, which occurs at menopause and persists until the end of life [3]. Estrogen replacement therapy has now become the preferential therapy in prevention of postmenopausal osteoporosis and should begin during the climacteric period and continue at least for 7–10 days [4]. However, due to the duration time of this therapy, one side effect, the increasing risk of endometrial cancer, has drawn much attention. In order to prevent osteoporotic bone fracture, maximizing the peak bone mass through appropriate nutrition has shown to be a good choice. Many researches have shown that prebiotics such as agave fructans [5], soybean whey [6], and soluble maize fiber [7] can promote calcium uptake. The corresponding mechanisms might be the production of short chain fatty acids, phytase enzymes, antioxidants and the reduction of intestinal inflammation [8]. Additionally, bioactive peptides are suitable candidates as supplements to improve calcium absorption in the gastrointestinal tract of the human body. Currently, calcium-chelating peptides have been isolated and characterized from various sources, including whey [9], alaska pollock backbone and skin [10], shrimp [11], chlorella [12], bovine serum [13], and wheat germ [14]. 

Some previous studies investigated the effect of peptides on the promotion of calcium uptake, bone formation, or osteoblast proliferation and differentiation. Casein phosphorpeptides (CPPs) have been reported to play an important role in intestinal calcium absorption and bioavailability in vitro [15,16]. CPPs could increase bone weight and calcium content [17] and prevent the bone loss in aged ovariectomized rats [18]. Moreover, some studies illustrated that CPPs could stimulate calcium uptake by primary human osteoblast-like cells and act as a direct modulator of bone cell activity [19,20].

Additionally, desalted duck egg white peptides (DPs) has been proven to promote calcium uptake in a calcium-deficient diet rat model [21] and a physic acid-induced calcium restricted mice model [22]. The corresponding mechanism was that DPs could interact with the transient receptor potential vanilloid 6 (TRPV6) calcium channel. Through the in vitro studies [23], it was clear that the main intestinal calcium absorption pathway was transcellular. However, it remains unclear whether DPs could promote bone formation or osteoblast proliferation and differentiation. Therefore, the primary aim of this research was to clarify the effect of DPs in the prevention of bone loss caused by retinoic acid and determine whether DPs modulate bone formation.

## 2. Materials and Methods

### 2.1. Preparation of Desalted Duck Egg White Peptides (DPs)

DPs with a molecular weight of less than 5 kDa were prepared from salted duck egg white as described in our previous work [21]. Briefly, salted duck egg white was desalted by electrodialysis equipment, followed by denaturation for 30 min in boiling water and cooling to 50 °C. The pH was adjusted to 6.5 prior to the addition of protamex (E:S = 1:25) and maintained at 6.5. After 3.5 h, the hydrolyzed solution was bathed in boiling water for 10 min to inactivate the enzyme. The degree of hydrolysis was 21.97%. Finally, the mixture was centrifuged at 3000× *g* for 10 min, and the supernatant was filtered through a hollow fiber membrane with a molecular weight cutoff of 5 kDa (PLCC, Millipore, Billerica, MA, USA). The filtrate was lyophilized and defined as DPs.

### 2.2. Animals and Experimental Design

The present study was conducted in accordance with the National Institute of Health Guidelines for the Care and Use of Animals and the animal ethical approval certificate number was MU-2016-001. Forty three-month-old Wistar female rats were obtained from Hubei Laboratory Animal Research Center and were maintained at the standard environmental conditions of 22 ± 2 °C, 60 ± 5% humidity, and 12 h dark/light cycle. The rats had free access to commercial food (Composition (g/kg): corn flour 500; wheat bran 90; wheat flour 90; bean dregs 220; fish flour 70; sodium chloride 5; multivitamins 1; trace elements 1.6; bone meal 20; calcium 7.501, magnesium 3.136, ferrum 0.276, zinc 0.055, and phytic acid 13.849) and tap water. After 1 week acclimation, the rats were divided into four groups: (i) healthy control group: rats received 0.9% saline for the whole experiment; (ii) model control group: rats received 80 mg/kg body weight retinoic acid (Shanghai Yuanye Biological Technology Company, Shanghai, China) once daily for the first 2 weeks as the osteoporosis model control and then 0.9% saline for other 3 weeks; (iii) positive control group: rats received 80 mg/kg body weight retinoic acid once daily for the first 2 weeks and then 5 mg/kg body weight alendronte (TOKYO Chemical Industry, Seoul, Korea) for other 3 weeks; (iv) rats received 80 mg/kg body weight retinoic acid once daily for the first 2 weeks and then 800 mg/kg body weight DPs for next 3 weeks. DPs and other reagents were given by gavage. At the end of week 4, the rats were housed individually, daily food intake was recorded and feces, urine were collected for the measure of calcium apparent absorption rate and calcium accumulation rate as follows: apparent Ca absorption rate (%) = (total Ca intake–Ca excretion in feces)/total Ca intake × 100%; accumulation Ca rate (%) = (total Ca intake–Ca excretion in feces–Ca excretion in urine)/total Ca intake × 100%. After the 5-week experiment, all the rats were fasted for 12 h and anesthetized by ether before sacrifice. Blood was collected from the ophthalmic venous plexus. Finally, each rat was sacrificed by cervical dislocation. Then, the femur and tibia were removed, weighed, and stored at −20 °C for further analysis. 

### 2.3. Serum Parameters

Serum calcium was determined by the methylthymol blue (MTB) assay. Briefly, calcium was combined with MTB in an alkaline solution to form a blue complex compound and determined at 610 nm. Serum phosphorus was determined by molybdenum blue method. Briefly, phosphorus was combined with molybdic acid to form phosphomolybdic acid which was then restored to molybdenum blue and determined at 660 nm. Serum bone alkaline phosphatase (BALP) and osteocalcin (OCN) were measured by ELISA kits. All the assay kits were purchased from Nanjing Jiancheng Bioengineering Institute (Nanjing, China).

### 2.4. Bone Parameters

The left femurs and tibias were thawed to room temperature before measuring the length with a digital caliper (Exploit, Beijing, China). Left femurs and tibias were analyzed using a texture analyzer system (TA. XT. PLUS, Stable Micro System, Godalming, Surrey, UK) and the bone was placed on a special holding device with two supports located at a distance of 20 mm with the large side up. Load was applied to the bone midpoint with a constant deformation rate of 10 mm/min until fracture occurred. The maximum load could be read directly from the force displacement graph recorded by the computer. After the stress test, the broken femurs and tibias were dried in an oven at 105 °C overnight and weighed on an analytical balance (ML204, METTLER TOLEDO, Zurich, Switzerland). Dry weight index was calculated according to the equation: DW (10^3^) = Dry weight × 1000/Body weight. Calcium content was measured by an atomic absorption spectrophotometer (AA6300C, Shimadzu, Kyoto, Japan). Bone mineral content (BMC) and bone mineral density (BMD) were determined by scanning the thawed right femurs and tibias with dual energy X-ray spectrometry (DEXA, XR-46, Norland, Fort Atkinson, WI, USA). The electron microscopy images of bones (middle section) were conducted by scanning electron microscope (JEOL JSM-6390/LV, NTC, Tokyo, Japan).

For tartrate-resistant acid phosphatase (TRAP) activity, the bones were immersed in 7% ethylenediaminetetraacetic acid (EDTA) at pH 7.2 for 5 days, the specimens were embedded in paraffin and serial sections 6 μm thick were obtained and then incubated for 1 h in 0.2 M sodium acetate buffer to pH 5.2 containing naphthol AS-BI (Sigma, St. Louis, MO, USA), Fast Red Salt (Sigma, St. Louis, MO, USA), and 50 mM sodium tartrate. After incubation, the sections were stained with hematoxylin. As controls for TRAP activity, some sections were incubated in a substrate-free medium. 

### 2.5. Establishment of Caco-2 Cell Monolayers

The Caco-2 cells were used as a model of the intestinal epithelium, and this cell line was purchased from the Cell Bank of Chinese Academy of Sciences (Shanghai, China). First, the cells were routinely grown in 75 cm^2^ plastic flasks (Greiner Bio-One GmbH, Frickenhausen, Germany) in minimum essential medium (MEM) (Gibco, Grand Island, NY, USA) with 15% fetal bovine serum, supplemented with 1% nonessential amino acids (Gibco, Grand Island, NY, USA) in the presence of 100 u/mL streptomycin and 100 u/mL penicillin. Next, the cells were incubated at 37 °C with 5% CO_2_. Cell passage numbers of 30–50 were used in this experiment. The culture medium was changed every 2 days until the flasks reached 90% confluence. 

At 90% confluence, the Caco-2 cells were detached with trypsin-EDTA treatment (final concentration of 0.005 g/L), followed by seeding the cells on 12-well millicell cell culture inserts (0.4 μm pore size, 12 mm diameter, Millipore Corporation, Billerica, MA, USA) with a cell density of 1.2 × 105 cells/mL and 21-day incubation. The volume of culture medium was 1.95 mL in the basolateral side and 0.4 mL in the apical side. Transepithelial electrical resistance (TEER) values were checked every 2 days by using a Millicell-ERS system (Millipore, Billerica, MA, USA) in order to evaluate the tight junction permeability of the Caco-2 cell monolayers. 

### 2.6. Calcium Transport Studies

The Caco-2 cell monolayers with TEERs exceeding 500 Ω·cm^−2^ were used for the calcium transport experiments. The monolayers were set in a 12-well plate and washed twice with warm (37 °C) balanced salt solution without calcium and magnesium (HBS) (125 mM NaCl, 4 mM KCl, 4 mM L-Glu, 10 mM Glucose, and 30 mM HEPES, adjusted to pH 7.4) and incubated at 37 °C for 20 min. The wells were then transferred to a new cluster plate containing 1.95 mL HBS buffer. Subsequently, 5 mmol/L calcium solution, 2-Aminoethydiphenylborinate (2-APB,50 μM), Nimodipine (30 μM), Val-Ser-Glu-Glu (VSEE) (synthesized by Hefei GuoTai Biology Co., Ltd., Hefei, China; 5, 10, 20 mmo/L), and DPs (2, 4, 8 mg/mL) were added to the apical side of the insert, and the calcium transport rates across Caco-2 cell monolayers were measured after 2 h. The TEERs were monitored before and after the addition of the calcium and DPs samples. Finally, the calcium contents were measured with an atomic absorption spectrophotometer (AA6300C, Shimadzu, Kyoto, Japan). 

### 2.7. Statistical Analysis

Experimental data were presented as mean values ± SD. The mean values were compared by Duncan’s multiple range test at *p* < 0.05 using SAS software version 8.1.

## 3. Results and Discussion

### 3.1. Physiological Variables and Apparent Absorption Rate

**Figure 1:**
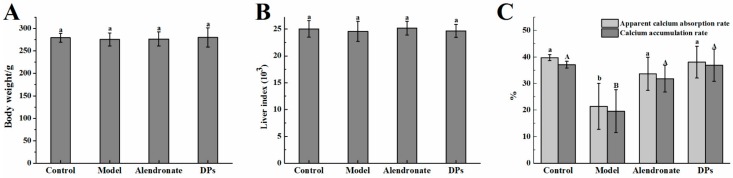
The effect of desalted duck egg white peptides (DPs) and alendronate on the (**A**) body weight, (**B**) liver index, and (**C**) apparent calcium absorption rate and calcium accumulation rate. ^a,b^ within column for apparent calcium absorption rate, means without a common letter are significantly different, *p* < 0.05; ^A,B^ within column for calcium accumulation rate, means without a common letter are significantly different, *p* < 0.05 (Duncan’s multiple range test at *p* < 0.05).

There is a list of medications exhibiting negative skeletal actions such as glucocorticoid and some anticonvulsant [24]. Retinoic acid is widely accepted as a medicine inducing osteoporosis and has been used to make a valuable and acceptable osteoporosis model for the ability to induce bone loss in a short period of time [25]. On the other hand, alendronate is widely used to treat osteoporosis [26]. In this study, rats were treated with retinoic acid (80 mg/kg body weight) to induce bone loss as a model group. DPs (800 mg/kg body weight) and alendronate (5 mg/kg body weight, as a positive control) treated rats were separately set as two treatment groups to compare indices related to bone loss and retention. To rule out adverse effects other than bone loss, body weight and liver index were tested among all the rats. As shown in Figure 1A,B, there were no significant differences observed in the body weight and liver index in each group (*p* > 0.05) after 5 weeks of experimentation, and no rats died during the experiment. However, the groups exhibited divergent results as related to calcium absorption. As shown in Figure 1C, retinoic acid had an inhibitory effect on the apparent calcium absorption rate and calcium accumulation rate (*p* < 0.05), but DPs and alendronate helped recover or prevent the adverse effects so that the levels of indices remained close to the control group. DPs and alendronate treatment groups showed almost no differences in calcium absorption and accumulation rates. 

This result indicated that the rats in each group were in the same physical condition and the test drugs in each concentration (80 mg/kg body weight retinoic acid, 800 mg/kg body weight DPs and 5 mg/kg body weight alendronate) had no adverse effects on rats apart from the calcium absorption. Retinoic acid inhibited calcium absorption significantly whereas DPs and alendronate seemed to reverse the results, which showed a positive sign. This result might be caused by retinoic acid, which could lower the rats’ appetite and then the rats absorbed less calcium than the control group. It was different from the calcium deficient diet animal model. The calcium apparent absorption rate and calcium accumulation rate in the model group was significantly higher than that in other groups [21]. 

Orsolic et al. (2014) reported the inverse results in that the rats’ body weight decreased after treatment with 80 mg/kg body weight retinoic acid and most rats died before the end of the experiment [2]. Nonetheless, no mortality and weight loss were observed in this study by retinoic acid treatment at the same dose. This different result could occur from the different treatment time. In the previous study, the test drugs (flavonoids) and retinoic acid were given once daily for 14 days and the animals could not stand the high toxicity and the body weight decreased. However, in this study, the rats received retinoic acid once for 2 weeks, then were treated with DPs or alendronate for another 3 weeks. 

Xu et al. (2005) reported that retinoic acid had good short-term effects on modeling osteoporosis, resulting in a decrease in cortical levels, an increase in osteoclasts numbers, an enhanced bone resorption, and the depression of endochondral ossification [27]. The effect of different dosages of Nylestriol/Levonorgestrel on bone metabolism in female rats with retinoic acid–induced osteoporosis was studied, and 0.15 mg/kg Nylestriol in combination with 0.015 mg/kg Levonorgestrel produced beneficial effects in bone metabolism. Retinoic acid induces bone loss by decreasing the estrogen level and increasing osteoclast numbers. It has been found that the retinoic acid receptor-related orphan receptors (RORs) are activated and the bone resorption rate increases [28]. One of the newly proposed mechanisms for the bone loss induced by retinoic acid is oxidative stress, which has been demonstrated in human and animal studies [29,30]. The rats treated with 80 mg/kg body weight retinoic acid resulted in a significant increase in MDA, which produces free radicals and measures of osteoclastic activity, indicating a state of increased oxidative stress [31]. 

As a positive control, it is now clear that alendronate inhibits bone resorption by affecting osteoclasts or osteoclast precursors in vivo. It has been reported that alendronate increases bone strength by increasing the mean degree of mineralization of bone tissue in osteoporotic women [3]. DPs in this study, was proved to have a similar prevention effect of bone resorption although the dose being used for DPs (800 mg/kg) was higher than that of alendronate. 

### 3.2. The Values of Calcium, Phosphate, BALP, and OCN in Serum

**Figure 2:**
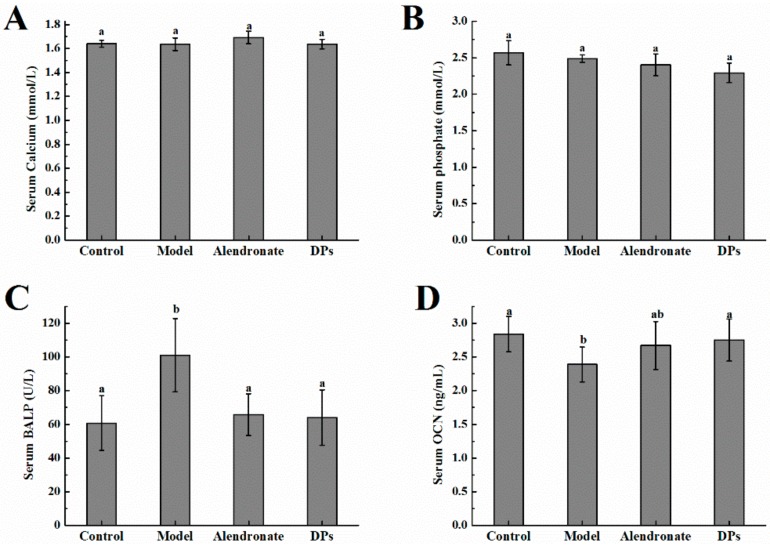
The effect of DPs and alendronate on the serum parameters. (**A**) Serum calcium content; (**B**) serum phosphate content; (**C**) serum bone alkaline phosphatase level (BALP); (**D**) serum osteocalcin level (OCN). ^a,b^ within column, means without a common letter are significantly different, *p* < 0.05 (Duncan’s multiple range test at *p* < 0.05).

There were no significant differences in serum calcium and serum phosphate in all groups (*p* > 0.05, Figure 2A,B). The bone alkaline phosphatase (BALP) level in the model group was higher than other groups (*p* < 0.05, Figure 2C). After treatment with alendronate or DPs, BALP decreased up to the normal level. BALP is one of the phenotypic markers of osteoblast activity, which can directly reflect the activity or functional status of osteoblasts. BALP is mainly used for the early diagnosis of infantile rickets, and it is the best indicator to evaluate human bone mineralization disorders. BALP level can be used to distinguish whether calcium uptake is deficient. After the treatment with retinoic acid, calcium levels in serum drop below the normal level. In order to maintain the calcium level, the calcium in bone is taken into the blood. 

The osteocalcin (OCN) levels in the model control group were significantly lower than that of the normal control and DPs groups (*p* < 0.05, Figure 2D). The rise of OCN is found in bone synthesis in clinic, especially in early bone synthesis after bone injury. The results indicate that the bone synthesis of rats in the model control group was not enough. After supplementation with DPs or alendronate, the synthesis rate increased and the effect of DPs was better than that with alendronate.

### 3.3. Bone Test Results

#### 3.3.1. Bone Calcium Content, Bone Length, Biomechanical Parameters, BMC, and BMD

**Figure 3:**
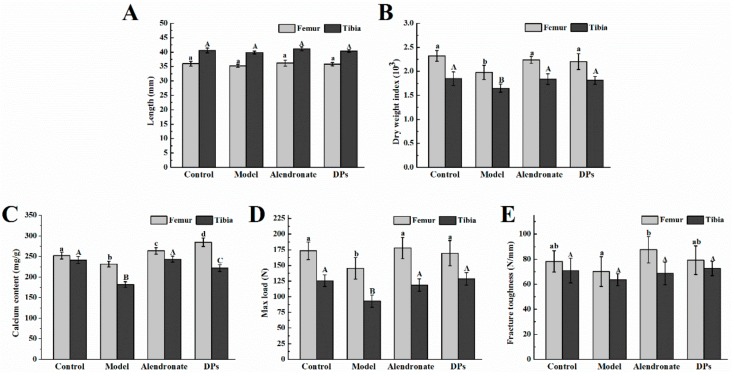
The effect of DPs and alendronate on the (**A**) bone length; (**B**) dry weight index; (**C**) bone calcium content; (**D**) max load of bone; and (**E**) fracture toughness of bone. ^a,b,c,d^ within column for femur, means without a common letter are significantly different, *p* < 0.05; ^A,B,C^ within column for tibia, means without a common letter are significantly different, *p* < 0.05 (Duncan’s multiple range test at *p* < 0.05). There was no statistical test performed between femur and tibia.

There were no significant differences in the length of femurs and tibias among all groups (*p* > 0.05, Figure 3A). The dry weight of the bones in the model group was significantly lower than that in other groups (*p* < 0.05, Figure 3B). After treatment with DPs and alendronate, the dry weight index increased, which confirmed their positive effects on bone synthesis. As shown in Figure 3C, the calcium content in bones in the model group was significantly lower than that in the other three groups (*p* < 0.05). After supplement with DPs and alendronate, the calcium content in femurs increased and was significantly higher than that of the control group (*p* < 0.05). To determine whether different treatments could improve bone strength, a three-point bending test of femurs and tibias was conducted as seen in Figure 3D,E. Femurs and tibias in the model group had lower maximum load and fracture toughness (*p* < 0.05). DPs and alendronate could both enhance the maximum load and fracture toughness to the normal level.

**Figure 4:**
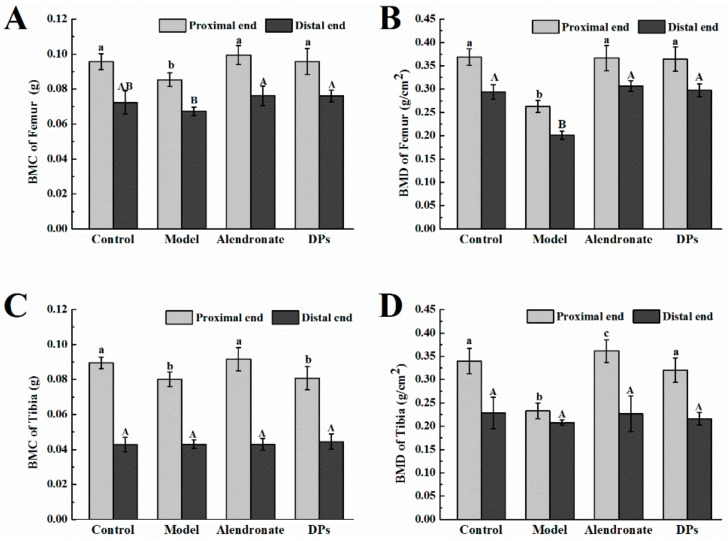
The effect of DPs and alendronate on the bone mineral content of femurs (**A**) and tibias (**C**), and bone mineral density of femurs (**B**) and tibias (**D**). ^a,b,c^ within column for proximal end, means without a common letter are significantly different, *p* < 0.05; ^A,B^ within column for distal end, means without a common letter are significantly different, *p* < 0.05 (Duncan’s multiple range test at *p* < 0.05). There was no statistical test performed between proximal ends and distal ends.

Bone mineral analysis also revealed consistent results. As shown in Figure 4A,B, the bone mineral content (BMC) and bone mineral density (BMD) of femurs in the model group were significantly lower than those in the other three groups (*p* < 0.05). After treatment with DPs and alendronate, BMC and BMD increased, except for the BMC of femurs in the distal end. The BMC of the tibias in the proximal end in the model and DPs groups was notably lower than that in the other two groups (*p* < 0.05, Figure 4C), and the BMD of the tibias in the proximal end in the model group was the lowest (*p* < 0.05, Figure 4D). There were no significant differences in the BMC and BMD of tibias in all groups (*p* > 0.05). Apart from the BMC and BMD tests, previous research applied a new method, quantitative microradiography, to access the mineral dimension of bone tissues and evaluate the bone strength and quality of bone and found that alendronate could increase bone strength by increasing the mean degree of mineralization of bone tissue in osteoporotic women [3].

#### 3.3.2. The SEM of Bones

The topography of mineralized bone surfaces could reflect the metabolic activity of bone cells, and it could be easily detected by scanning electron microscopy (SEM). There are three distinct topographies about the mineralized bone surfaces by SEM [32]. Firstly, bone formation by osteoblasts consist of the production and mineralization of a complex extracellular matrix exhibited by knobby projections. Secondly, bone resorption by osteoclasts results in a confined, localized removal of mineral and matrix, and resorbing bone surfaces seen by SEM are scalloped. Thirdly, a smooth bone surface indicates a region undergoing neither formation nor resorption. In a growing skeleton, most bone surfaces are undergoing formation or resorption, and they will appear either knobby or scalloped by SEM [33]. This morphologic method can have wide application which facilitates rapid, accurate assessment of skeletal metabolism. 

**Figure 5:**
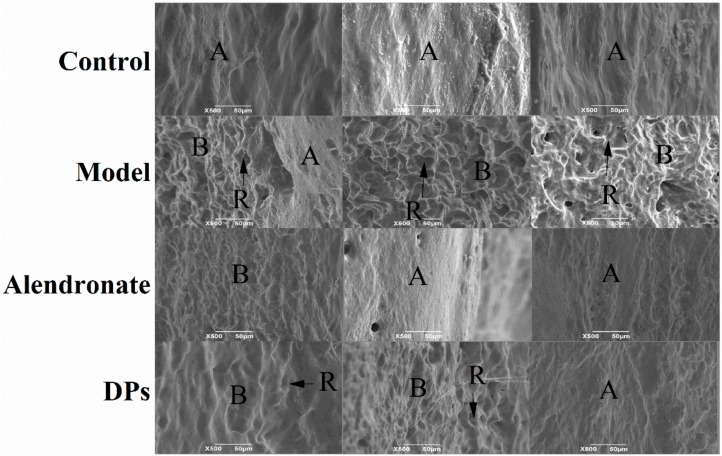
Scanning electron micrograph of bones in each group. Three pictures of each group are identified by surface topography and reveal the metabolic state of the bone. **Control:** smooth section (**A**) neither formation nor resorption was evident. **Model:** scalloped section (**B**) bone resorption and individual resorption lacunae (**R**) were evident. **Alendronate:** smooth section (**A**) and scalloped section (**B**) both existed and fewer resorption lacunae (**R**) than model group were observed. **DPs:** smooth section and scalloped section both existed and resorption lacunae were more evident compared to that in the Alendronate group.

In the present study, SEM was used to make a comparison in the effect of DPs and alendronate on the metabolic activity of bone. As shown in Figure 5, the bone surfaces in the control group were smooth, which indicated that the bones were stable and underwent neither formation nor resorption and were covered by squamous bone lining cells or flattened osteoblasts. In the model group, the bone surfaces were scalloped due to bone resorption by osteoclasts. After treatment with alendronate and DPs, the scalloped regions decreased significantly compared to the model group. 

#### 3.3.3. The Tartrate-Resistant Acid Phosphate (TRAP) Staining of Bones

**Figure 6:**
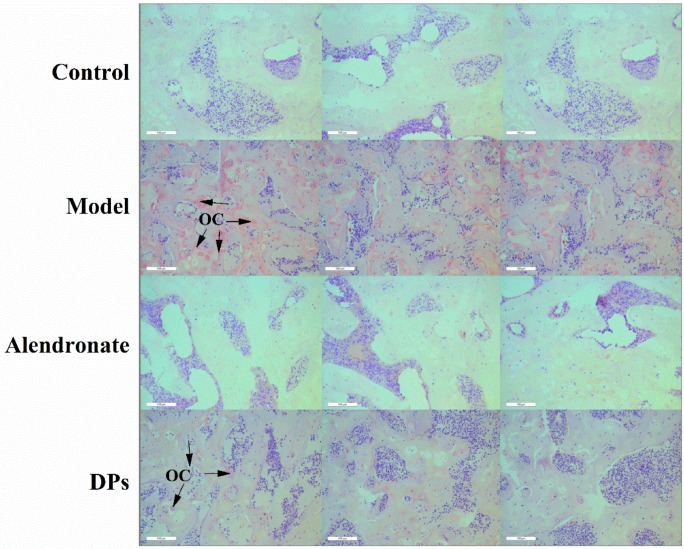
The tartrate-resistant acid phosphatase (TRAP) activity in each group. The cytoplasm of osteoclasts (OC) is wine red and the nucleus is blue. **Control:** the wine red section was not evident. **Model:** the wine red area increased compared to the control group, and osteoclasts (OC) are labelled (arrow). **Alendronate:** the wine red section was not as evident as in the control group. **DPs:** the wine red area decreased compared to the model group, and osteoclasts (OC) are labelled (arrow). Bar = 100 µm.

The tartrate-resistant acid phosphate (TRAP) method is a useful method to measure the activity of osteoclasts. Some researchers combined terminal deoxynucleotidyl transferase-mediated dUTP-biotin nick end labeling assay (TUNEL) and TRAP methods to facilitate visualization of the relationship between osteoclasts and apoptotic bone cells during alveolar bone remodeling [34]. After the staining steps, the cytoplasm of osteoclasts is wine red and the nucleus is blue. As seen in Figure 6, the wine red sections in the model group were significantly larger than other groups, which indicated that the activity of osteoclasts in the model group was higher. Retinoic acid induces bone loss by decreasing the estrogen level and increasing osteoclasts numbers. After treatment with alendronate and DPs, the number of osteoclasts decreased and the effect of alendronate was better than that of DPs. 

**Figure 7:**
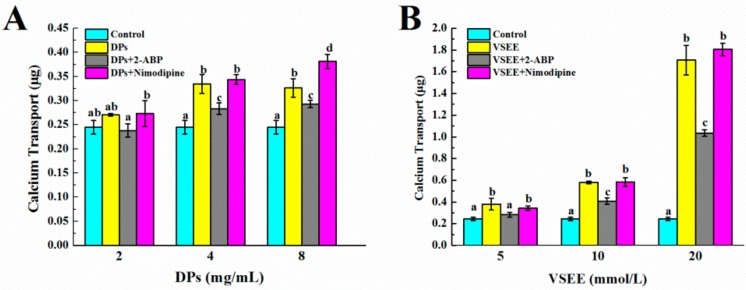
The effect of DPs ((**A**); 2, 4, 8 mg/mL) and Val-Ser-Glu-Glu peptide (VSEE) ((**B**); 5, 10, 20 mmol/L) on the calcium transport in the presence of 100 µM 2-aminoethoxydiphenyl borate (2-APB) or 30 µM Nimodipine. ^a,b,c,d^ within a DPs or VSEE concentration, means without a common letter are significantly different, *p* < 0.05 (Duncan’s multiple range test at *p* < 0.05).

As shown in Figure 6 and Figure 7, the bone resorption rate was slowed down and the numbers of the osteoclasts decreased in rats treated with DPs compared to that in the model group. Taken together, these results indicate that DPs might have the same function as CPPs as a modulator of bone cell activity. Although the exact mechanism has not been clarified, it can be considered as a potential anabolic factor for bone tissue engineering. 

### 3.4. The Pathway of Calcium Absorption by DPs

There are two main pathways of intestinal calcium absorption: one transcellular, the other paracellular. The transcellular transport consists of three steps: (1) the entrance of Ca^2+^ across the brush border membranes of enterocytes through epithelial Ca^2+^ channels TRPV6, TRPV5, and Cav1.3; (2) Ca^2+^ movement to the basolateral membranes by binding proteins such as Calbindin D9k; and (3) Ca^2+^ extrusion into the blood by the plasma membrane Ca^2+^ ATPase pump (PMCA1b) or sodium calcium exchanger (NCX1) [35]. It has been verified that DPs enhance calcium transport and may do so by acting as calcium carriers and interacting with the cell membrane, whereas the paracellular pathway may only make a minor contribution [22,23]. Structure characterization demonstrated the important roles of some crucial peptides, such as Val-Ser-Glu-Glu peptide (VSEE), in binding calcium and promoting calcium uptake. Since the transcellular pathway is the major way of calcium uptake by DPs and VSEE, it is necessary to evaluate the exact calcium channel. 

It has been reported that the human epithelial calcium channel TRPV6 could be inhibited by 2-aminoethoxydiphenyl borate (2-APB) using fluorescence imaging, patch clamp, and radioactive tracer techniques in transiently and stably transfected HEK293 cells [36]. Nimodipine, as an antagonist, was able to totally or partially reduce the CPP-induced calcium rise in HT-29 RPMI cells [37]. As shown in Figure 7A, 4 mg/mL and 8 mg/mL DPs could increase calcium transport significantly in Caco-2 cell monolayer models (*p* < 0.05) in comparison with that of the control group. The calcium transport decreased significantly (*p* < 0.05) after the treatment of 100 µM 2-ABP in the presence of 4 and 8 mg/mL DPs. Thirty µM Nimodipine did not show the same result as 2-ABP and in the presence of 8mg/mL DPs with 30 µM Nimodipine, the calcium transport was notably higher (*p* < 0.05) than the groups with 8 mg/mL DPs without Nimodipine. The calcium transport increased significantly in the presence of 5, 10, and 20 mmol/L VSEE (*p* < 0.05, Figure 7B). After the addition of 2-ABP, the calcium transport decreased significantly (*p* < 0.05). Also, the addition of Nimodipine did not show a decrease of calcium transport. This result confirmed the hypothesis that DPs improved calcium uptake through the TRPV6 channel. However, it could not be clarified whether the Cav1.3 calcium channel was the transport pathway of DPs in this study. 

Caco-2 cells, mainly of absorptive types, are similar to the enterocytes of the duodenum [38]. In the duodenum, where active transport occurs, the TRPV6 channel is predominant [39]. Unlike Caco-2 cells, HT-29 cells are primarily heterogeneous types with a predominance of goblet type and resemble the cells residing in the ileum region. The L-type calcium channels are physiologically present in enterocytes, with a more elevated expression in distal jejunum and proximal ileum [40]. The absorption of calcium is conventionally considered to be higher, about 85–90%, and utilizes the paracellular route [40]. This might be the reason that Nimodipine did not inhibit the calcium transport in Caco-2 cell monolayers.

## 4. Conclusions

DPs and alendronate can improve calcium absorption rate, bone dry weight, bone calcium content, max load, bone mineral content, and density in the retinoic acid-induced bone loss model of rats. The surface topography of bone results showed that these two substances could increase bone formation and inhibit bone resorption. The histology results indicated that the osteoclast numbers decreased under the treatment of DPs and alendronate. The in vitro study indicated that the TRPV6 calcium channel was the intestinal calcium uptake pathway by DPs and VSEE. Apart from the calcium-deficient diet rat model and physic acid-induced calcium restriction mice model, this research expands the understanding of the mechanism of cellular calcium uptake by DPs in vitro, and DPs might act as a modulator on bone formation and bone resorption in vivo. In the future, the Ovariectomized (OVX) rat model, which is a known animal model for postmenopausal osteoporosis, will be used to explore the exact mechanism on bone formation by DPs.
